# No Influence of Overweight/Obesity on Exercise Lipid Oxidation: A Systematic Review

**DOI:** 10.3390/ijms21051614

**Published:** 2020-02-27

**Authors:** Avigdor D. Arad, Anthony J. Basile, Jeanine Albu, Fred J. DiMenna

**Affiliations:** 1 Division of Endocrinology, Department of Medicine, Icahn School of Medicine at Mount Sinai, New York, NY 10029, USA; avigdor.arad@mssm.edu (A.D.A.); ajbasile@asu.edu (A.J.B.); Jeanine.AlbuMD@mountsinai.org (J.A.); 2 Department of Biobehavioral Sciences, Columbia University Teachers College, New York, NY 10027, USA

**Keywords:** exercise lipid oxidation, overweight/obesity, insulin signal transduction pathway, insulin resistance, type 2 diabetes, intramuscular triglycerides

## Abstract

Compared to lean counterparts, overweight/obese individuals rely less on lipid during fasting. This deficiency has been implicated in the association between overweight/obesity and blunted insulin signaling via elevated intramuscular triglycerides. However, the capacity for overweight/obese individuals to use lipid during exercise is unclear. This review was conducted to formulate a consensus regarding the influence of overweight/obesity on exercise lipid use. PubMed, ProQuest, ISI Web of Science, and Cochrane Library databases were searched. Articles were included if they presented original research on the influence of overweight/obesity on exercise fuel use in generally healthy sedentary adults. Articles were excluded if they assessed older adults, individuals with chronic disease, and/or exercise limitations or physically-active individuals. The search identified 1205 articles with 729 considered for inclusion after duplicate removal. Once titles, abstracts, and/or manuscripts were assessed, 24 articles were included. The preponderance of evidence from these articles indicates that overweight/obese individuals rely on lipid to a similar extent during exercise. However, conflicting findings were found in eight articles due to the outcome measure cited, participant characteristics other than overweight/obesity and characteristics of the exercise bout(s). We also identified factors other than body fatness which can influence exercise lipid oxidation that should be controlled in future research.

## 1. Introduction

Obesity [1] and type 2 diabetes (T2D) [2] are prevalent conditions. The insulin resistance (IR) that often predates T2D is linked with overweight/obesity; however, the mechanistic basis(es) underpinning this association is/are unclear with multiple candidate molecules, systems, and pathways potentially involved [3]. One theory implicates dysfunctional lipid metabolism based on the notion that excess accumulation of lipid in skeletal muscle (intramuscular triglyceride; IMTG) becomes ‘lipotoxic’ and perturbs the insulin signal transduction pathway [4]. A growing body of recent research provides further insight.

### 1.1. The Influence of Elevated Intramuscular Triglycerides on the Insulin Signal Transduction Pathway

**Figure 1:**
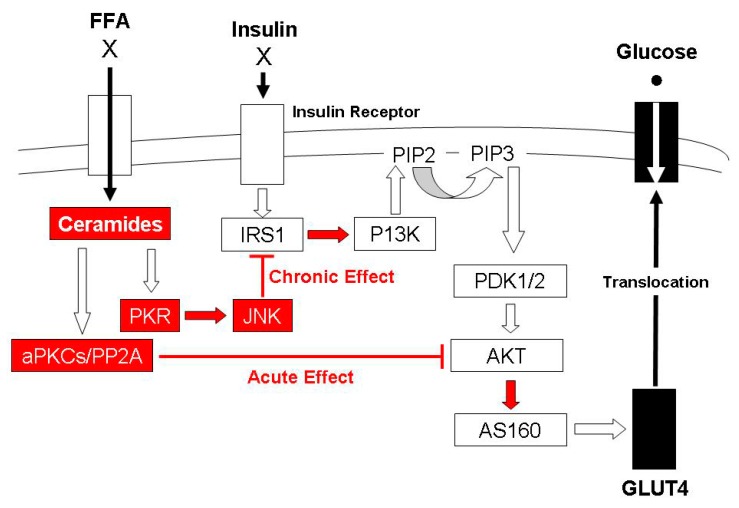
Schematic representation of the deleterious influence of elevated intramuscular ceramide concentration on two different steps of the insulin signal transduction pathway. The acute effect involves ceramide-induced inhibition of protein kinase B (Akt) via protein phosphatase 2 (PP2A) or atypical protein kinase Cs (aPKCs) while a chronic effect occurs due to activation of the protein kinase R (PKR) stress pathway which negatively impacts insulin receptor substrate 1 (IRS1) [8].

It has long been known that IMTG levels are inversely related to insulin action [5]; however, the presence of elevated IMTG in endurance-trained athletes with high insulin sensitivity (the ‘athlete’s paradox’) indicates more than a simple cause-effect relationship [6]. Contrary to their experimental hypothesis, Amati et al. showed that exercise-trained muscle does not possess lower levels of diacylglycerols (DAG), a potentially-harmful lipid intermediate that was believed to be one of the causative links between elevated IMTG and the blunted insulin signaling that is present with obesity [7]. Indeed, for normal-weight athletes, total DAG content, saturated DAGs and DAG species in which one of the fatty acids was unsaturated was ~50% and ~200% higher compared to the levels that were found in the skeletal muscle of normal-weight sedentary and obese individuals, respectively [7]. Conversely, the total content of the sphingolipid metabolite ceramide and both the saturated and unsaturated subdivisions were greater in obese skeletal muscle compared with the two normal-weight groups [7]. More recently, it has been shown that treatment of myotubes with ceramide activates the apoptosis-inducing protein kinase R (PKR) stress pathway such that short-term exposure results in inhibition of protein kinase B (Akt) while prolonged treatment inhibits insulin-mediated insulin receptor substrate 1 (IRS1) activation [8]. Consequently, elevated ceramide has the potential to interfere with insulin action at two steps in the signal pathway that is responsible for the translocation of glucose transporters that stimulate glucose uptake by skeletal muscle [8]. Figure 1 (adapted from [8]) provides a schematic representation of this series of events. Consistent with this contention, 12 weeks of supervised exercise training that increased peripheral insulin sensitivity for individuals with obesity and normal glucose tolerance or obesity and T2D decreased fasting plasma C14:0, C16:0, C18:1, and C24:0 ceramide levels with the change in both total and C14:0 associated with the observed improvement in insulin-stimulated glucose disposal [9]. Collectively, these findings lend credence to the contention that it is the concentration of ceramides in muscle that is responsible for the link between elevated IMTG and the insulin resistance that is often observed with obesity [10].

### 1.2. The Cause of Increased Accumulation of Lipotoxic Metabolites with Overweight/Obesity

Dietary saturated fatty acid intake profoundly influences hepatic synthesis and serum concentration of ceramide [11]; hence, an association between overweight/obesity and the pathological accumulation of ceramide is not surprising [12]. Interestingly, acute exercise also increases serum ceramide concentration; however, in this case, values return to basal level during recovery [13]. Both cross-sectional [14,15] and longitudinal [16,17] analyses indicate that a chronic adaptation to endurance training is an increased ability for lipid oxidation at a given rate of work. It, therefore, seems reasonable to conclude that the athlete’s paradox can be attributed to the fact that contrary to pathological accumulation, the elevated IMTG observed in trained individuals is a positive metabolic adaptation designed to place a readily available fuel source in proximity to where it is routinely relied upon. Conversely, there is evidence to suggest that obese individuals demonstrate impaired lipid oxidation that could be responsible for nonfunctional and, indeed, pathological accumulation of IMTG [18,19]. Importantly, this observation has been made during fasting [18,19], a condition with energetic demand that should be satisfied predominantly by lipid as substrate. However, on the other hand, assessing lipid use during fasting is questionable because of the low metabolic demand and the prevailing influence of acute energy balance and dietary macronutrient consumption [20]. A better alternative might be to investigate lipid use during moderate-intensity exercise when metabolic activity is higher, yet lipid can still contribute significantly [20]. The exercising condition might also be superior for exploring the degree to which a reduced capacity for lipid oxidation contributes to elevated IMTG because there is a greater relative contribution of skeletal muscle tissue to whole-body lipid oxidation during exercise compared to rest.

### 1.3. The Influence of Overweight/Obesity on Exercise Lipid Oxidation

Obese individuals possess reduced mitochondrial content [21] and lower oxidative-enzyme capacity [22], ‘downstream’ factors that could limit oxidation of available lipid during exercise. Moreover, ‘upstream’ limitations in lipid availability during exercise might be present with overweight/obesity due to a greater cortisol response [23], impaired growth hormone [23] and/or plasma catecholamine [24] responses, and/or reduced lipolytic effect of a given level of circulating catecholamines [25]. There is also evidence of a blunted exercise-induced reduction in insulin concentration with obesity [26]. However, despite this theoretical justification for an association between an obesity-related impairment in lipid use and lipid metabolite accumulation that perturbs the insulin signal transduction pathway, studies performed to test the hypothesis that the reliance on lipid during exercise is reduced with overweight/obesity have returned equivocal findings. There is even evidence of enhanced exercise lipid use with obesity, which might reflect increased availability of IMTG [27,28] and/or substrate selection influenced by IR that often accompanies excess body-fat accumulation [29]. The reason(s) for conflicting findings regarding the influence of overweight/obesity on exercise lipid use is/are unclear, but might reflect methodological differences regarding important factors that should be controlled to isolate the influence. For example, fat-free mass (FFM) and cardiorespiratory fitness (CRF) of the participant [27] and energy balance and macronutrient composition of their diet [30,31] affect exercise lipid use irrespective of body-fat stores. The ability to use lipid is also influenced by the intensity [32,33] and duration [32] of the exercise bout.

Resolving ambiguity regarding the influence of overweight/obesity on lipid oxidation during exercise is important because understanding this influence can provide insight into the aetiology of the disease. For example, if a deficiency is present in overweight/obese individuals, it is consistent with the contention that an inherent defect (e.g., compromised lipolytic activity that limits lipid availability and/or mitochondrial dysfunction that restricts lipid oxidation) predisposes the overweight/obese condition [26,33]. In theory, a shift in substrate from lipid to carbohydrate during physical activity would be accompanied by a reciprocal shift toward lipid storage, the more energetically-efficient way to increase energy stores when a positive energy balance is present. Consequently, reduced lipid oxidation might be associated with a greater propensity to gain body fat [34,35]. Moreover, the degree to which overweight/obesity affects exercise lipid oxidation and the influence it exerts might be important factors to consider when prescribing exercise to manage/treat the disease [27]. For example, if impaired lipid oxidation accompanies the overweight/obese state, exercise for improving mitochondrial function (e.g., high-intensity interval training) would be warranted [36,37]. A higher-intensity approach (e.g., sustained exercise at a metabolic rate exceeding that which allows for the maximal lipid-oxidation rate) would also be advantageous for overweight/obese individuals because the ‘target energy expenditure’ to ensure adequate rate of weight loss [38] can be achieved with reduced time commitment. Conversely, if lipid use is not reduced with overweight/obesity, an exercise metabolic rate at which lipid oxidation is maximal (e.g., at ‘FATmax’) might be preferred to improve metabolic health irrespective of body-fat loss by optimally perturbing the IMTG pool [39,40] and/or changing its relationship with muscle mitochondria [41]. This approach would also cohere with the recent emphasis placed on increasing step count with less attention paid to the intensity of effort being endured while taking those steps. However, an uncompromised capacity for exercise lipid oxidation with overweight/obesity is not consistent with the contention that obesity-dependent IR is due to impaired mitochondrial function in skeletal muscle [19].

The primary aim of this systematic review was to formulate a consensus regarding the influence of overweight/obesity on exercise lipid use from existing research. A secondary aim was to inform future research by exploring important factors other than body fatness that must be controlled to isolate this influence.

## 2. Results

**Figure 2:**
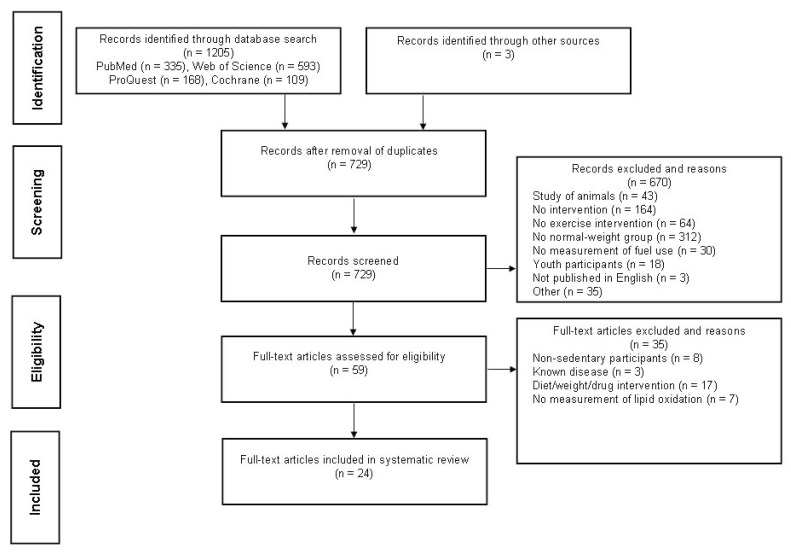
Flow diagram depicting the study-selection process that was used to conduct this systematic review.

Figure 2 illustrates the flow diagram for this systematic review. The search identified 1205 articles with 729 considered for inclusion after duplicate removal. Once titles, abstracts, and/or manuscripts were assessed, 24 satisfied inclusion criteria.

Table 1 presents information about the included studies.

All of the included investigations were cross-sectional in nature; however, three also included longitudinal components involving energy restriction [48,49] or endurance training [41]. For the former, the lipid-oxidation comparison between lean and obese participants occurred at baseline only [48,49] while the latter involved comparisons before and after the intervention [41].

In 17 studies, a comparison of whole-body [23,26,27,28,41,42,43,44,46,47,58,59] or whole-body and source-specific [26,27,28,53] lipid oxidation during exercise between individuals who do not possess excess body fatness (most often called ‘lean,’ but also referred to as ‘control,’ ‘leaner,’ ‘never obese,’ ‘nonobese’, and ‘normal weight’) and overweight/obese individuals was a primary purpose. In two of these, the effect of weight loss was also investigated by recruiting obese individuals who had lost weight; however, a third group comprising obese individuals who had not lost weight was also included [26,46]. For the other seven studies, one investigated the effect of time of day on exercise lipid use with normal and obese participants compared during morning and evening [54] while the other six assessed differences due to participant characteristics other than overweight/obesity with a comparison between normal-weight and overweight/obese participants also provided. For example, one compared lean and obese African American with lean and obese Caucasian women; however, a two-way ANOVA was conducted to determine the independent effect of obesity both within and across race [34]. Two studies investigated whether exercise substrate use differs for individuals with T2D; however, lean and obese individuals comprised two healthy control groups so that the effect of obesity without the influence of T2D was also reported [45,52]. Two other studies explored the relationship between free fatty acid (FFA) availability and lipid oxidation for obese individuals with different body-fat distribution; however, a nonobese group was also included for comparison [48,49]. Finally, one study assessed endurance-trained individuals to determine 24-h substrate use in trained lean compared to untrained obese individuals on exercise and nonexercise days [50]. However, untrained lean individuals were also included and a comparison of substrate use exclusive to the exercise bout was provided.

### 2.1. Methodological Characteristics of Included Studies

#### 2.1.1. Stratification Criteria for Body Fatness

In 13 studies, BMI was used exclusively to stratify participants [26,27,34,41,42,43,49,50,51,52,53,54,55,56,57,59]. In two of these, the World Health Organization (WHO) classification system was used to define ‘normal’ and ‘overweight’ (BMI, <25 and ≥25 kg∙m^−2^, respectively) [43,55] while in another, the cutoff point was arbitrary (28 kg∙m^−2^) [34]. Additionally, in one study, the WHO system was used to stratify three groups by differentiating obese (BMI, ≥30 kg∙m^−2^) from overweight [53] while in another, a BMI of 30 kg∙m^−2^ was used as a threshold to differentiate ‘obese’ from ‘control’ [42]. We chose to include the latter study because the mean ± SD for the control group was 20.9 ± 0.5 kg∙m^−2^ (*n* = 8). In the other eight studies that used BMI exclusively, participants were classified into zones to provide separation; for example, obese [41,50,54,57] or extremely-obese [26] compared to normal-weight using the WHO criterion (‘extremely obese’ BMI, ≥35 kg∙m^−2^) or obese compared to individuals with BMI <23 [56] or <24 [27,49] kg∙m^−2^.

In four studies, body-fat percentage was used exclusively for stratification with various criteria employed; for example, obese and lean designations as >25% and <16%, respectively [23], an obese/lean cutoff point of 30% for women [58,59] and 20% for men [59] or age-specific (20–39 or 40–59 yrs) overweight/normal-weight cutoff points for women (32.5% and 34.5%) and men (20.5% and 22.5%) [47]. In two other studies, body-fat percentage was used with BMI; for example, obese participants possessing body fat >30% with BMI >29 kg∙m^−2^ compared to ‘never-obese’ controls with body fat no more than ‘slightly above 30%’ and BMI <27 kg∙m^−2^ (mean ± SD, 20.6 ± 0.9 kg∙m^−2^; *n* = 5) [46] or obese with body-fat percentage >40% and BMI of 35.0–39.9 kg∙m^−2^ compared to lean with body-fat percentage <30% and BMI ≤23 kg∙m^−2^ [28]. Finally, in five studies, the mean ± SD for BMI [45] or both BMI and body-fat percentage [44,48,51,52] for each group was provided to illustrate differences with no mention of a criterion employed.

#### 2.1.2. Dietary Control

In 14 studies, participants exercised in the fasted state with no longer-term dietary control [23,26,34,41,42,43,44,47,52,54,55,57,58,59]. Five other studies included greater short-term control; for example, in addition to an overnight fast, in two studies, participants exercised after a standardized meal [28] or meal plus snack [53] the evening before while in three others, a standardized breakfast was provided prior to testing [46,50,56]. In seven studies, longer-term control was in place. In two cases, participant-specific diets were prescribed; specifically, a eucaloric diet with set macronutrient proportions for three (energy content based on three-day recall) [46] or four (energy content estimated based on FFM with activity factor) [50] days plus breakfast prior to testing. The other five studies with longer-term control included the same generic diet for all participants; for example, a ‘balanced diet’ comprising the same macronutrient and energy content for 21 or four days for obese and lean individuals, respectively [51], or a diet containing ≥200 g of carbohydrate daily for three days [27,45,49] or ‘at least two weeks’ [48] prior to an overnight fast before testing.

With respect to the ‘fasted state,’ it was defined in four studies as the condition during the morning following an overnight fast [27,28,42,45] while in others, a timeframe was provided; for example, 4 [23], 4–6 [58], 5–6 (evening session) [54], 8 [34,57], 8–12 (morning session) [54], 10–12 [48,52,53], 12 [26,41,47,49,51,55], or 15 [43] h. In two other studies, the ‘fasted’ [44] or ‘post-absorptive’ [59] state was not defined. Finally, in the three studies that required exercise following breakfast, the time interspersed was 60 [50], 90 [46], or 180 [56] min.

#### 2.1.3. Criterion Exercise Challenge

Table 1 also presents characteristics of the criterion exercise bout(s) used to assess substrate selectivity. In 23 studies, endurance exercise was studied; specifically, conventional leg cycle ergometry [23,26,27,34,41,42,45,46,47,48,49,50,51,52,55,59], recumbent cycling [28,53], treadmill exercise [43,49,54,57,58], vacuuming (MET level >3; hence, included) [47], floor walking [47], platform stepping [47], or mode not stated [56]. In one of these, in addition to leg ergometry, participants performed arm cranking for comparison across muscles groups [52]. The one study that did not involve what would be considered endurance exercise required circuit resistance training with sets performed intermittently for 10–12 repetitions at 70%–75% of the one-repetition-maximum weight [44].

Of the 23 studies on endurance exercise, 13 required one testing session during which a single continuous bout at a constant work rate (CWR) was performed for 30 [23,49,57], 40 [45], 60 [26,27,41,46,50], 90 [28,53], or 150 [48] min, or duration required so that energy expenditure reached 300 kilocalories for each participant (~30 and ~28 min for lean and obese, respectively) [56]. Another study also involved CWR during a single session; however, two 10-min bouts at different work rates were performed in succession [34]. Six ‘activities of daily living’ were done in succession with 2 min of recovery between in another study [47]. For this review, only the final four (vacuuming, floor walking, platform stepping, and leg cycling) were considered ‘exercise’ (reported MET level >3) and they were performed continuously for 12, 6, 12, and 12 min, respectively. In two others, participants performed two CWR bouts on separate days for 15 [58] or 45 [43] min. In these two studies, fuel use was also assessed during a third session using an incremental ‘Bruce protocol’ (3 min stages in succession) that was ‘open ended’; i.e., continued until termination criteria were achieved (e.g., heart rate of 220 minus age, ventilatory equivalent for O_2_ ‘close to 30 l∙min^−1^, and RER >1.15 [43]) or at least two of the following: A plateau in VO_2_, RER ≥1.1, rating of perceived exertion ≥18, and/or ‘volitional fatigue’ [58]. Incremental protocols were used exclusively in the other six investigations comprising endurance exercise; however, in two of these, close-ended paradigms were employed. Specifically, one required four 6-min bouts in succession [55] while another involved four (women) or five (men) 5-min bouts performed with 5 min of rest interspersed [59]. The other four investigations involved open-ended tests with two utilizing 3-min stages until an RER of 1.0 [54] or limit of tolerance [42] and another comprising 6-min stages until a criterion RER (>1.0) or power output (>65% of previously-determined peak) was achieved [51]. The fourth investigation involving open-ended incremental testing included both a leg and arm protocol performed until limit of tolerance on separate days. For the leg test, stage length was set at five minutes until an RER of 1.0 was achieved after which 2-min stages were done [52]. For the arm test, three 6-min stages were completed and a 5-min rest period allowed before 1-min stages performed until limit of tolerance [52].

In 14 of 17 studies involving assessment of substrate use during CWR, work rate was calculated relative to the VO_2peak/max_ achieved during a maximal incremental test; for example, 40% [45], 45% [48], 50% [26,27,28,41,47,53,58], 55% [50], 60–65% [46], 65% [34], 70% [49], or 75% [57,58]. One of these involved a two-bout series with the first-bout work rate set at the same absolute power output (15 W) for all participants [34]. One of the other three CWR studies involved walking and running at 1.0 km∙h^−1^ below and above the preferred walk/run transition speed [43] while in another, vacuuming, floor walking, and platform stepping were regulated by participants at their ‘usual pace’ [47]. In the other two CWR investigations, work rates were aligned with the ‘ventilatory threshold’ estimated using the VCO_2_-VO_2_ relationship [56] or ventilatory-equivalent-for-CO_2_ response [23] from an incremental test.

A number of intensity-loading paradigms were used in the eight studies that assessed substrate use during incremental exercise. Six involved increments prescribed in the same absolute terms for all participants; for example, in two employing treadmill exercise, a modified Bruce Protocol starting at 2.7 km∙h^−1^ with 0% [43] or 5% [58] was used. Another study required treadmill walking at 3.5 km∙hr^−1^ at a 1% grade followed by speed increases of 1.0 km∙h^−1^ for four stages and grade increases of 2% thereafter [54]. Three other studies involved stages at the same absolute work rate during cycle ergometry; specifically, 0-W leg cycling followed by 30-W increments [42,59] or exercise at 95 or 20 W followed by work-rate increments of 35 or 15 W for leg and arm cycling, respectively [52]. Conversely, the other two studies comprising incremental exercise involved increments established relative to participant capacity; for example, individuals cycled at 20% of their peak work rate measured previously on an incremental test followed by work-rate increments of 7.5% per stage [51] or an initial work rate that was 20% of the estimated peak with four increases of 10% per stage thereafter [55].

### 2.2. Conclusions from Included Studies Regarding the Influence of Overweight/Obesity on Exercise Lipid Use

Table 1 presents findings regarding exercise lipid use for included studies and outcome measure(s) cited to draw the conclusions. Thirteen studies provide no evidence of altered exercise lipid oxidation for overweight/obese compared to normal-weight individuals [23,26,41,42,43,45,46,48,50,53,56,57,58]. Outcome measures indicating no difference in exercise lipid oxidation in these studies were: (1.) RQ/RER [23,26,41,42,45,46,50,58]; (2.) total lipid oxidation in absolute terms [46,48,56], relative to body mass and FFM [46], and/or as a percentage of total energy expenditure [45,56]; (3.) lipid-oxidation rate in absolute terms [43,48,57] or relative to body mass [26,41,45] and/or FFM [41,53]; and (4.) maximal lipid-oxidation rate in absolute terms, relative to FFM and as an RER, heart rate, VO_2_ relative to body mass, and VO_2_ as a percentage of VO_2max_ [43].

The conclusion that there is no difference in exercise lipid oxidation for overweight/obese individuals was drawn from four of nine studies on men exclusively [23,43,53,56], six of nine on women exclusively [26,41,42,46,48,58], and three of six on a mixed group [45,50,57]. The conclusion was supported by 12 of 17 studies involving CWR endurance exercise [23,26,41,43,45,46,48,50,53,56,57,58] and three of eight for which incremental endurance exercise was assessed [42,43,58]. No difference in exercise lipid oxidation between overweight/obese and normal-weight individuals was found in nine of 17 studies involving leg cycling [23,26,41,42,45,46,48,50,53], three of five involving treadmill exercise [43,57,58] and one study for which mode was not stated [56].

Seven studies included evidence of decreased lipid oxidation for overweight/obese compared to normal-weight individuals for some [34,47,51,54,59] or all [44,55] outcome measures, populations, and/or modes assessed. Outcome measures indicating decreased exercise lipid oxidation in these studies were: (1.) RER [34,44,55,59]; (2.) total lipid oxidation as a percentage of energy expenditure [47]; (3.) lipid-oxidation rate in absolute terms [34,47,51,55] and/or relative to FFM [34,51,54,55,59]; (4.) maximal lipid-oxidation rate expressed as a power output [55], percentage of VO_2peak/max_ [51,54], WR_max_ [55], and/or HR_max_ and/or as a zone [51]; and (5.) lipid/carbohydrate crossover point in absolute and relative terms as a work rate and percentage of WR_max_, respectively [55].

The conclusion that there is reduced exercise lipid oxidation for overweight/obese individuals was drawn from three of nine studies on men exclusively [44,51,54], one of nine on women exclusively [34] and three of six on a mixed group [47,55,59]. The conclusion was supported by two of 17 studies involving CWR endurance exercise [34,47] and four of eight involving incremental endurance exercise [51,54,55,59]. Reduced exercise lipid oxidation for overweight/obese individuals was found in four of 17 studies involving leg cycling [34,51,55,59], one of five involving treadmill exercise [54] and the single studies on resistance training [44] and physical activities of daily living [47]. Finally, two studies indicated population specificity with respect to reduced exercise lipid oxidation with overweight/obesity; for example, for females, but not males [59] and for females when compared to Caucasian, but not African American normal-weight controls [34].

Five of 24 studies included evidence of increased lipid oxidation for overweight/obese compared to normal-weight individuals for some [27,49,51,52] or all [28] outcome measures and/or populations assessed. Outcome measures indicating increased exercise lipid oxidation in these studies were: (1.) RER [27,49,51]; (2.) lipid energy expenditure as a percentage of total energy expenditure [27,28]; (3.) lipid-oxidation rate in absolute terms [51] and/or relative to FFM [28,49,51]; and (4.) maximal lipid-oxidation rate as a percentage of VO_2peak/max_ [52] and as an RER [51].

The conclusion that there is increased exercise lipid oxidation for overweight/obese individuals was drawn from three of nine studies on men exclusively [27,51,52] and two of nine on women exclusively [28,49]. The conclusion was supported by three of 17 studies involving CWR endurance exercise [27,28,49] and two of eight for which incremental endurance exercise was assessed [51,52]. Greater exercise lipid oxidation for overweight/obese compared to normal-weight individuals was found in three of 17 studies involving leg cycling [27,28,51], one of five involving treadmill exercise [49] and the only study on arm cranking [52].

Eight of 24 studies returned equivocal findings based on multiple outcome measures, populations, and/or exercise characteristics assessed (see Table 2).

Seven of 17 studies including report of multiple outcome measures provided evidence that choice of outcome measure can influence whether the null hypothesis is accepted or rejected [27,34,47,49,51,52,54]. Interestingly, two of three studies including report of sex-specific results indicate that an overweight/obesity-related decrement is present for males, but not females [47,59] and a similar disparity was identified for African American compared to Caucasian women in the only study to compare races [34]. Furthermore, findings from one of two studies comprising two groups of obese individuals based on fat-deposition pattern suggested that this factor can influence the conclusion at least based on one of two outcome measures that was reported [49]. Finally, findings from one of three studies assessing lipid oxidation at different time points during CWR exercise [49], four of nine investigating different intensities/work rates [34,51,54,59] and both studies analyzing different exercise modes [47,52] showed that each of these characteristics of the exercise session can influence the conclusion that is drawn.

### 2.3. Quality Assessment of Included Studies

Quality-assessment scores of included studies are provided in Table 3. The mean ± SD score for methodological quality was 1.51 ± 0.14 out of 2.0 (median, 1.46; range: 1.29–1.79).

## 3. Discussion

The purpose of this systematic review was to form a consensus regarding the degree to which overweight/obese individuals rely on lipid as fuel during exercise compared to normal-weight counterparts. This is important because a decreased capacity to use lipid as fuel is one explanation that has been advanced to explain the association between overweight/obesity and insulin resistance because the pathological accumulation of lipid metabolites (e.g., ceramide) in muscle perturbs the insulin signal transduction pathway [4]. However, we found that some or all outcome measures in 21 of 24 included studies indicate that the null hypothesis regarding an influence of overweight/obesity on exercise lipid use cannot be rejected (Table 1). This means that the preponderance of evidence suggests that overweight/obese and nonoverweight/obese individuals rely on lipid to a similar extent during exercise. However, eight of these 21 studies also include report of a significant difference in exercise lipid use for overweight/obese individuals based on exercise characteristics, participant characteristics other than overweight/obesity and/or outcome measures assessed (Table 2). A likely conclusion is that more research is needed to clarify what is, in all likelihood, a multifactorial, complex influence of overweight/obesity on exercise metabolism.

### 3.1. Rationale for and Evidence of Decreased Exercise Lipid Oxidation for Overweight/Obese Individuals

Despite similar arterial FFA concentration and absolute skeletal-muscle rate of FFA uptake, obese individuals demonstrate lower rates of lipid oxidation during fasting when lipid use should predominate compared to normal-weight counterparts [19]. This implies a ‘downstream’ limitation that has been attributed to defective mitochondrial structure/function [21,22] which might also be responsible for excess body-fat gain [34,35] and the association between obesity and IR via elevated IMTG [19]. Interestingly, lean children with a severely-obese parent demonstrate impaired lipid oxidation during cycling compared to counterparts with nonobese parents [60], which coheres with the contention that the defect is inherent. Consistent with this line of reason, obese individuals should demonstrate impaired lipid oxidation during exercise; however, only seven of 24 included studies provide any evidence which supports this contention [34,44,47,51,54,55,59]. Furthermore, in one of these studies, a higher RER for obese compared to lean men was observed during intermittent resistance exercise, an activity during which lipid would not be expected to contribute to a great extent in healthy individuals [44]. Alternatively, five studies indicating an obesity-related decrement involved comparison during stationary cycling, which is important because this is a modality typically recommended as low-impact exercise to reduce body fat and improve metabolic health for overweight/obese individuals [34,47,51,55,59]. However, in four of these studies, the decrement was only present under certain conditions. For example, while obese participants demonstrated a lower lipid-oxidation rate per FFM at higher intensities and experienced their maximal lipid-oxidation rate at a lower relative work rate, their lipid use at moderate intensities and lipid-oxidation rate at the apex of the lipid-oxidation/exercise-intensity curve was not different compared to lean counterparts [51]. Moreover, obese participants demonstrated lower RER and higher rates of lipid oxidation at low intensities in that study. This discrepancy exemplifies the importance of paying careful attention to the method used to normalize intensity when assessing exercise metabolism (see below).

Participant characteristics other than overweight/obesity can also play a role in lipid oxidation during exercise. For example, obese African American and Caucasian women demonstrated a higher RER during cycling at 15 W; however, this was only the case when lean Caucasian women comprised the control group [34]. Conversely, when obese women of both races were compared to lean African American women, no difference was present. This implies that some genotypes and/or phenotypes convey a lipid metabolic defect regardless of body fatness. Furthermore, there was also no difference in exercise RER for obese women of both races compared to lean African American women when participants cycled at the same relative intensity (65% VO_2peak_); however, in this case, only Caucasian obese women possessed a decrement compared to their lean Caucasian counterparts. In addition to participant characteristics, this again exemplifies how the method used to assign intensity can affect findings. More evidence of potential confounding influences comes from Keim et al. (1996), who had participants perform an incremental cycling test at the same absolute work rates and found that overweight/obese men had a higher RER when cycling at 120 W compared to leaner men [59]. However, no differences were present for men at four other work rates or for women at any work rates performed. These researchers also used each participant’s VO_2_/work-rate relationship to estimate substrate-oxidation rates at 30%, 40%, 50%, and 60% VO_2max_ and found that lipid oxidation increased for leaner men as intensity increased whereas for overweight/obese men, it decreased. Consequently, lipid-oxidation rate, which did not differ between groups at 30% VO_2max_, was significantly lower for overweight/obese men at 40%, 50%, and 60% VO_2max_. Conversely, there was no difference at the four estimated work rates for women in that study. With respect to sex, these findings cohere with those of Grams et al. (2016) who report that overweight men use less lipid compared to lean counterparts when cycling at 50% VO_2max_ while overweight women do not [47]. However, during self-paced stepping, which was also assessed in that study, both sexes experienced an overweight-related decrement [47]. Finally, in the one study indicating an obesity-related decrement during treadmill exercise, obese men demonstrated lower lipid oxidation during incremental exercise at higher work rates relative to VO_2max_, but lipid use at lower work rates and the maximal lipid-oxidation rate were not different compared to normal-weight controls [54]. Collectively, these findings suggest that if a decrement in exercise lipid use is present with overweight/obesity, identification can depend on the mode and intensity of the criterion exercise challenge and the race and sex of the participant.

Of the 23 studies involving endurance exercise, only one provides unmitigated evidence that exercise lipid use is reduced for overweight/obese individuals. Perez-Martin et al. (2001) stratified participants of both sexes into normal and overweight groups and found that overweight individuals demonstrated a higher RER and lower rate of lipid oxidation for all stages of an incremental cycling protocol comprising 6-min bouts at 30%, 40%, 50%, and 60% of their estimated maximal work rate [55]. Furthermore, the maximal lipid-oxidation rate and the ‘crossover point’ from lipid to carbohydrate occurred at lower relative work rates for the overweight individuals. In this study, these researchers also confirmed that when similar 6-min bouts were performed on separate days, ‘steady-state’ VO_2_ and VCO_2_ were not different compared to the incremental values, which suggests that the sequential nature of the protocol did not affect substrate-oxidation rates. However, the degree to which 6 min of exercise at a specific work rate is sufficient to achieve substrate-oxidation rates that faithfully reflect an individual’s ‘true’ capacity for lipid oxidation at that work rate (and, therefore, a capacity that is valid for comparison across different types of individuals) has been questioned [49,58].

### 3.2. Rationale for and Evidence of Increased Exercise Lipid Oxidation for Overweight/Obese Individuals

While it is attractive to speculate an inherent mitochondrial defect that adversely influences lipid use and predisposes overweight/obesity and IR, in vivo measurements indicate that exercise-stimulated whole-body lipid oxidation might not be impaired when a decrease in mitochondrial density is present in normal-weight healthy men [61] or by the reduction in mitochondrial content [52,62] and/or function [63,64] that is present with T2D. Consequently, it might not be surprising that we found weak evidence for an overweight/obesity-related decrement in exercise lipid use. However, there are reasons why overweight/obese individuals might demonstrate *greater* reliance on lipid during exercise compared to normal-weight counterparts. Firstly, it is intuitive that greater fat mass in adipose tissue could provide for greater plasma FFA availability in response to exercise. However, a review of literature suggests an obesity-related downregulation of lipolysis per quantity of fat mass [65] and reduced lipolytic response to exercise for overweight/obese individuals [53] that offsets the advantage. Alternatively, the ‘window’ for lipid contribution might be widened by IR that often accompanies obesity, which is linked to decreased muscle-glycogen use during exercise [29]. Finally, IR is accompanied (and possibly caused by) elevated IMTG [66] and the associations between obesity, elevated IMTG, and IR might imply alterations in accumulation of lipid in muscle and blunting of insulin action that conspire to create a different pattern of exercise fuel use for overweight/obese individuals with IR compared to normal-weight individuals without it. Four included investigations that also involved stable-isotope-tracer infusion to determine the source of oxidized lipid during exercise provide interesting insight. In three studies [27,28,53], the contribution of nonplasma lipid (presumably predominantly IMTG) was increased with obesity while in the other [26], an increase of ~15% did not reach statistical significance. However, this enhanced ability to use IMTG for obese individuals only allowed for greater whole-body lipid oxidation in two of these studies [27,28]. In the other two, obese individuals demonstrated a concomitant reduction in the ability to oxidize plasma FFA such that a similar whole-body lipid-oxidation rate was observed compared to normal-weight counterparts [26,53]. Consequently, mixed conclusions regarding the degree to which exercise lipid use is increased, decreased, or unaffected for overweight/obese individuals might be attributable at least in part to a difference in the net effect of changes in nonplasma and plasma contributions of lipid with some overweight/obese individuals able to maintain the capacity for plasma lipid use along with greater use of IMTG. The reason(s) for such a disparity is/are unclear, but might involve the degree to which lipolytic activity and/or insulin action is reduced in association with obesity. For example, in addition to an increased reliance on nonsystemic fatty acids and decreased reliance on systemic fatty acids, Mittendorfer et al. (2004) found an inverse relationship between adiposity and the exercise lipolytic response relative to fat mass that they linked to reductions in peak plasma epinephrine concentration and adipose-tissue catecholamine sensitivity [53]. The end result was that unlike lean controls, it was FFA availability in plasma instead of uptake by muscle that rate limited plasma lipid oxidation in overweight/obese individuals. However, other studies do not support this limitation. For example, Lanzi et al. (2014) found reduced lipolytic activity at all work rates during incremental exercise for obese males; however, their increased quantity of adipose tissue compensated for the deficit allowing for greater FFA availability during exercise compared to lean counterparts and, by extension, greater and unaffected lipid-oxidation rates at low and moderate intensities, respectively [51]. Moreover, Horowitz et al. (2000) found similar whole-body lipolytic activity for women with abdominal obesity compared to lean controls [28] while Goodpaster et al. (2002) report ~50% greater rate of FFA uptake during exercise for obese males [27].

The aforementioned findings suggest a complex interplay regarding the capacity to maintain plasma FFA availability/uptake/oxidation alongside an obesity-related increase in IMTG availability/oxidation that ultimately dictates the net effect on whole-body lipid oxidation. Factors that might influence this interplay are sex, fat-deposition pattern, and/or degree of obesity; however, the degree to which IR is present and has progressed might be the most important one to consider. Blaak et al. (2000) observed similar total lipid oxidation for individuals with T2D compared to healthy controls matched for body composition during moderate-intensity cycling although the individuals with T2D demonstrated increased reliance on triglyceride-derived lipids to compensate for lower exercise-induced rate of appearance/oxidation of plasma FFA [67]. This contrasts findings by Braun et al. (2004), who report a similar FFA availability/use profile along with increased total lipid-oxidation rate during moderate-intensity cycling for overweight/obese normoglycemic women with IR compared to women without IR matched for body composition [29]. Although the source of oxidized lipid was not assessed in that study, it seems reasonable to speculate that increased reliance on IMTG to fill the void created by deceased reliance on muscle glycogen in association with the initial stages of IR had not yet been offset by the decrease in plasma lipid oxidation that ultimately accompanies the progression of IR to T2D [67].

### 3.3. Individual Characteristics that Might Influence Exercise Lipid Use Irrespective of Body Fatness

#### 3.3.1. Sex

Only three of six included studies on both males and females accounted for the potential influence of sex by reporting sex-specific results either exclusively [47] or in addition to collectively [55,59]. All three provide evidence that obese males oxidize lipid less effectively during exercise compared to normal-weight counterparts [47,55,59] while two indicate this is not the case for obese females [47,59]. Greater susceptibility to an obesity-related decline in exercise lipid use for males resonates because similar sexual dimorphism in exercise metabolism has been confirmed for normal-weight individuals [68,69,70,71]. The reason(s) for an enhanced capacity for lipid oxidation for females is/are unclear, but might relate to increased β- [70] and/or decreased α-adrenergic [72,73] activity resulting in greater FFA circulation/uptake [68,73], greater uptake of a given level of circulating FFA [71], and/or greater storage/use of IMTG [71]. Regardless of this distinction, greater lipid oxidation for females raises the possibility that females possess inherent characteristics that allow them to fend off a decline in the ability to oxidize lipid that accompanies overweight/obesity. In support of this contention, eight of nine included studies comprising females exclusively suggest an unchanged [26,41,42,46,48,58] or enhanced [28,49] exercise lipid oxidation for overweight/obese compared to normal-weight counterparts. However, for nine included studies comprising males exclusively, only three indicate a reduction with obesity [44,51,54]. To clarify ambiguity regarding the influence of sex on exercise fuel use with overweight/obesity, future research should include both males and females with results analyzed according to sex.

#### 3.3.2. Race

African Americans demonstrate reduced lipid oxidation at rest [74,75], during exercise [74], and when challenged with a high-fat diet [76]. However, only one included study controlled for the influence of race when comparing exercise lipid use for overweight/obese compared to normal-weight individuals. Hickner et al. (2001) observed higher RER and lower lipid-oxidation rate for obese African American and Caucasian women when cycling at 15 W; however, this was only the case when lean Caucasians represented the controls [34]. Conversely, the ~30% lower lipid-oxidation rate demonstrated by lean African American compared to Caucasian women during the bout was quantitatively similar to that observed in obese individuals of both races [34]. The reason(s) for reduced lipid oxidation for African Americans is/are unclear, but might relate to reduced insulin sensitivity and mitochondrial capacity [77] and/or a lower proportion of type I muscle fibers along with greater activity of ‘anaerobic’ metabolic enzymes [78]. Regardless of this distinction, a decrement in exercise lipid use for normal-weight African Americans suggests that races should be studied separately when investigating the influence of body fatness on exercise metabolism.

#### 3.3.3. Fat-Free Mass

Overweight/obese individuals typically possess greater FFM compared to lean counterparts. For example, FFM was measured/reported in 16 included studies and in all but two [47,48], FFM was greater with overweight/obesity; *c.f.,* for females, but not males [59], obese, but not overweight individuals [53] and upper-, but not lower-body obesity [49]. This is important because if a comparison of lipid oxidation is made using an absolute measurement (e.g., lipid-oxidation rate in g∙min^−1^ or µmol∙min^−1^ and/or total lipid oxidation in grams, µmol or kilocalories), the value should be expressed relative to FFM because it is influenced by the amount of metabolically-active tissue the individual possesses [27]. Conversely, if relative measurements (e.g., RER or lipid use as a percentage of energy expenditure) are cited, no adjustment is required. In [19] included studies, absolute measurements were recognized as some [26,27,34,41,43,45,46,47,49,51,55,56,59] or all [28,48,52,53,54,57] outcome measures and in eight cases, some [43,47,52,56] or all [26,45,48,57] were stated with no adjustment for FFM. Interestingly, in seven of these eight studies, no difference was reported when comparing overweight/obese and normal-weight individuals with unadjusted measures [26,43,45,48,52,56,57]. This is consistent with the contention that failure to express an absolute measurement relative to FFM can cause a type II error by masking an obesity-related decrement. However, in three of these seven studies, relative measurements were also reported which indicated similar exercise lipid use regardless of body fatness [26,45,56] while in two others, support was provided by values of the maximal lipid-oxidation rate, which were normalized to FFM even though rates for individual stages of the test were not [43,52]. Alternatively, for 11 studies that included a report of total lipid oxidation or lipid-oxidation rate as a function of FFM, only four indicate no difference in the adjusted measurement between groups [27,41,46,53]. For the other seven, one provides conclusive evidence that lipid oxidation is reduced for overweight/obese individuals [55] while four suggest that it can be the case, but only under certain circumstances; for example, in Caucasian, but not African Americans [34], for males, but not females [59] and during exercise at higher, but not lower intensities [51,54]. Finally, the two other studies that included a report of lipid-oxidation rate as a function of FFM indicate that the adjusted rate is greater for overweight/obese individuals [28,49]. This ambiguity likely reflects the confounding effect(s) of other variables that can influence exercise lipid use irrespective of body fatness. Nevertheless, in future research, authors should recognize the influence of FFM on absolute measurements of lipid use and control for this factor when comparing overweight/obese and normal-weight individuals.

#### 3.3.4. Aerobic Fitness

Endurance-trained individuals demonstrate enhanced lipid oxidation during exercise [79] due to greater reliance of nonplasma sources [14] and longitudinal studies confirm reduced carbohydrate reliance and increased lipid use during endurance exercise at the same absolute work rate after chronic endurance training [80,81]. It has, therefore, been suggested that to properly test the hypothesis that exercise lipid use is altered with overweight/obesity, lean and obese individuals must be matched for ‘aerobic fitness’ [27]. However, only eight included studies made mention of such matching [27,28,41,46,48,51,53,59] and specific information of how matching was accomplished was only provided in one case (i.e., pair-wise range matching for VO_2peak_ per FFM; range, ±3 mL∙kg^−1^∙min^−1^) [59]. Furthermore, all eight studies used VO_2peak/max_ as the proxy measure, which could be problematic because the aspect of aerobic fitness that rate limits lipid oxidation is likely different than that which rate limits O_2_ consumption. Specifically, for a given level of FFA availability, lipid oxidation is predominantly dictated by peripheral factors (e.g., mitochondrial mass and oxidative enzyme activity) which allow a metabolic rate to be sustained with/without perturbation of mitochondrial phosphorylation and redox potentials [82]. Conversely, VO_2peak/max_ is generally limited by convective and/or diffusive steps along the O_2_-transport cascade [83]. It is, therefore, not surprising that training-related changes in mitochondrial capacity can be dissociated from changes in VO_2peak/max_ [84]. Interestingly, Ara et al. (2011) report that the peak lipid-oxidation rate occurs at a higher work rate relative to VO_2peak/max_ in obese and post-obese individuals compared to controls despite similar VO_2peak/max_ values [85]. Collectively, these findings suggest that the aspect of aerobic fitness that should be matched to properly assess whether lipid use is altered with overweight/obesity and is related to muscle metabolic as opposed to central circulatory capacity and a parameter that reflects this capacity is the highest metabolic rate that can be sustained without perturbation of cellular phosphorylation and redox states (i.e., the metabolic rate at the ‘lactate threshold; VO_2_LT). The influence of differences in VO_2_LT for individuals matched according to V_O2peak/max_ can be accounted for when normalizing exercise intensity across participants (see below).

#### 3.3.5. Insulin Resistance

Excess adiposity, particularly in the abdominal region [86], is often accompanied by IR, which means that the capacity for insulin-mediated glucose uptake by skeletal muscle is blunted. While IR does not adversely affect contraction-mediated glucose uptake [87], overweight/obese individuals with IR rely less on muscle glycogen and more on lipids during exercise compared to BMI-matched individuals without IR [29]. Interestingly, it has been suggested that evolutionary preservation of IR indicates its ‘usefulness’ for protecting survival in circumstances where glucose sparing takes precedence (e.g., acute trauma and prolonged fasting) [88]. Collectively, these observations raise the intriguing possibility that exercise lipid reliance might be altered with IR irrespective of body fatness and if this is the case, a comparison of exercise lipid use between overweight/obese and normal-weight individuals could be confounded by the difference in insulin responsiveness that would typically be present.

Interestingly, 12 of 14 included studies containing a report of fasting plasma insulin concentration confirmed a higher value in overweight/obese participants [26,27,28,41,42,45,46,48,51,52,55,57]. However, no attempt was made to control for IR in any studies included in this review. Indeed, only one included mention of its potential influence with Goodpaster et al. (2002) reporting that despite relative hyperinsulinemia, fasting insulin was not associated with exercise fatty-acid oxidation for the obese individuals they assessed [27]. This implies that IR was not responsible for the higher contribution of lipid to exercise energy expenditure they observed for obese participants. Nevertheless, it seems reasonable to suggest that future research designed to determine how exercise lipid use is influenced by overweight/obesity should include matching of the IR status of the groups (e.g., by recruiting normal-weight controls with IR that matches overweight/obese participants or recruiting overweight/obese participants without IR).

#### 3.3.6. Menstrual Phase

As part of its role as primary female sex hormone, estrogen improves numerous aspects of energy homeostasis including insulin sensitivity and catecholamine-stimulated lipolysis [89] with several studies confirming increased exercise lipid use during the luteal phase of the menstrual cycle when circulating estrogen is high [90,91,92]. However, only four of nine studies comprising females exclusively involved control for this factor [28,46,48,49] and, interestingly, two of these report greater exercise lipid use for overweight/obese compared to normal-weight women [28,49]. Importantly, both assessed females during the follicular phase when the cyclical increment should be absent [91]. Conversely, five investigations that involved exclusive study of women without controlling for menstrual phase provide no evidence for an overweight/obesity-related increment [26,34,41,42,58]. One possible explanation is that a propensity for greater exercise lipid oxidation associated with overweight/obesity in women is only identifiable when controls are not experiencing their cyclical elevation. Consequently, much like what has been recommended for sex-comparative studies [93], it seems reasonable to suggest that comparisons of exercise metabolism between overweight/obese and normal-weight women should be performed in both phases of the menstrual cycle to elucidate the effect of overweight/obesity on exercise lipid use with both low and high circulating estrogen.

#### 3.3.7. Fat-Deposition Pattern

While it is typical to refer to a generic influence of overweight/obesity on many aspects of physiology, there is growing appreciation that the influence can be markedly different depending upon where fat is situated. For example, greater visceral and ectopic deposition and a lower percentage localized subcutaneously in the gluteofemoral region are patterns associated with IR and dyslipidemia [94,95]. This resonates with respect to exercise fuel use because a key factor believed to underpin the disparity is the degree to which lipolysis is stimulated in these regions by circulating epinephrine [96]. Interestingly, it has been shown that normal-weight women with lower abdominal-to-lower-body fat-mass ratios possess higher maximal rates of lipid oxidation during incremental exercise compared to women with higher proportions of central fat [97]. Moreover, compared to normal-weight women with a high abdominal-to-lower-body fat-mass ratio, normal-weight women with a lower ratio mobilize and oxidize more lipid when cycling at 65% VO_2max_ in association with differences in circulating hormones related to lipolysis (e.g., growth hormone, insulin, and atrial natriuretic peptide) [98]. Considering these findings, fat-deposition pattern is another variable that could confound a comparison of exercise lipid use for overweight/obese compared to normal-weight individuals. However, two included studies stratified overweight/obese participants according to fat-deposition pattern and results suggest that the disparity in exercise lipid oxidation reported for normal-weight women with upper-body adiposity compared to lower-body adiposity [97,98] is not extant for obese individuals [48,49] even though a difference in the exercise-induced increase in FFA was observed [48]. This implies that overweight/obese individuals with different fat-deposition patterns can be grouped together when comparing exercise fuel use with normal-weight counterparts. However, given the aforementioned findings for normal-weight individuals, it still might be necessary to control for this factor to decrease the likelihood of type II error by reducing the variability in criterion measurement of lipid use for normal-weight controls against which overweight/obese individuals are compared.

#### 3.3.8. Acute and ‘Long-Term’ Dietary Habits

Dietary habits profoundly influence substrate use. For example, Black et al. (1986) demonstrated that the macronutrient ratio of food intake (i.e., the ‘food quotient’; FQ) approximates RQ for individuals in energy balance [99] while Miles-Chan et al. (2015) confirmed that the principal exogenous factor that influences post-absorptive RQ is dietary FQ [100]. Specifically, they report a positive relationship between percent dietary carbohydrate intake and post-absorptive RQ under both normo- and under-/overfeeding conditions with an increase from ~0.80 to ~0.90 in response to increased carbohydrate ingestion from 30% to 60% of total energy intake [100]. Importantly, research confirms that fluctuating carbohydrate [30] or fat [31] content of the diet also influences RQ during exercise, which implies that a controlled-feeding period should be imposed prior to assessing exercise lipid use. However, unlike carbohydrate oxidation, which adapts rapidly in response to changes in carbohydrate intake [101,102], lipid oxidation responds slowly when a high-fat diet is implemented [103,104] with a time course further lengthened by obesity [105] and family history of T2D [106]. Being that a balanced controlled diet might reflect a change in lipid intake for some participants, this implies that dietary control prior to exercise testing should be more extensive than a simple acute manipulation such as testing in the fasted state or the fasted state following ingestion of a standardized meal. It is also important to recognize that energetic intake should be closely matched to expenditure during any controlled-feeding period because lipid is the predominant substrate used/stored in response to short-term fluctuations in energy balance [101]. Finally, following any period of dietary control, to ensure that the exercise metabolic response will not be affected by the final meal of the regimen, participants should be tested after a sufficient period of fasting (e.g., >6 h) [107].

Given the influence of feeding on substrate oxidation, it is surprising that only seven included studies involved more than acute dietary control [27,45,46,48,49,50,51]. Moreover, in four of these seven, a general guideline regarding a minimum amount of carbohydrate intake was all that was provided [27,45,48,49] while in two others, participants were not tested in the fasted state after completing their controlled-feeding period [46,50]. Hence, acute and long-term dietary control with macronutrient standardization in energy balance was only enforced in one included study and, unfortunately, results from this study are inconclusive because obese individuals oxidized lipid more, less or equally as effectively as normal-weight controls during exercise depending upon outcome measure considered [51]. Future studies designed to clarify the influence of overweight/obesity on exercise lipid use should include control of both macronutrient composition and energy balance for an extended period to ensure weight stability with similar prevailing FQ.

### 3.4. Exercise Variables that Might Influence Exercise Lipid Use Irrespective of Body Fatness

#### 3.4.1. Mode

The modes of exercise most often used for exercise testing are treadmill walking/running and leg-cycle ergometry [108]. Consequently, it is not surprising that 22 of the 24 included studies involved one of these modalities (Table 1). However, in one of these studies, arm cranking was also performed so that differences in substrate use in arm compared to leg muscle could be explored [52]. Specifically, citing research indicating that arm muscles retain a better capacity for glucose clearance in older hypertensive individuals [109] and individuals with T2D [110], Larsen et al. (2009) hypothesized that the influence of T2D on exercise metabolism would be different during arm compared to leg cycling [52]. However, in addition to lean controls against which exercise substrate use by individuals with T2D was compared, a group of healthy obese participants was also included; hence, a comparison of exercise lipid oxidation for healthy obese compared to normal-weight individuals in arm compared to leg muscle was made [52]. Interestingly, the maximal lipid-oxidation rate during incremental exercise occurred at a higher percentage of VO_2max_ for obese compared to lean participants during arm, but not leg cycling [52]. While neither lipid-oxidation rates at the variety of intensities investigated nor the maximal rate were different between obese and lean individuals, this suggests that testing upper-body musculature might allow an obesity-related increment in exercise lipid use to be unveiled. The reason(s) for different metabolic activity in arm compared to leg muscle is/are unclear, but might relate to muscle phenotype [52] because a high proportion of type I fibres provides a greater ‘window’ for the loss of insulin prowess that occurs with T2D [111]. Collectively, these findings are consistent with our contention that the state of IR is an important factor to control when assessing exercise metabolism for overweight/obese compared to normal-weight individuals (see above). Furthermore, they indicate that future studies of exercise metabolism with overweight/obesity should include both leg and arm ergometry (in particular, utilizing the CWR exercise model).

#### 3.4.2. Duration

During prolonged moderate-intensity exercise, muscle-glycogen reliance decreases while the whole-body rate of lipid oxidation increases in association with increased availability/oxidation of plasma FFA [32,112]. If the time course of this upregulation is different for overweight/obese compared to normal-weight individuals, duration of an exercise bout used to assess exercise metabolism must be selected accordingly. Interestingly, Kanalay et al. (2001) observed a similar lipid-oxidation rate for obese and nonobese women after 15 min of treadmill walking; however, for the obese women, the rate increased from 15 to 30 min whereas for nonobese women, it did not [49]. The end result was that 30 min of exercise required the same energy expenditure, but 30% more lipid oxidation for obese women [49]. Unfortunately, these researchers terminated exercise at 30 min; hence, it cannot be determined whether nonobese participants would have eventually ‘caught up.’ Nevertheless, this suggests that longer exercise duration is required to reveal an obesity-related increment that might be present and findings by Horowitz et al. (2000) provide further support as whole-body lipid oxidation from 60 to 90 min of cycling was ~25% greater for obese compared to lean women [28]. Conversely, Goodpaster et al. (2002) report that the lower RER they observed for obese compared to lean men at 30, 45, and 60 min of cycling was also present at 15 min [27]. Moreover, other than the three studies mentioned above, the other 10 that involved assessment of lipid oxidation for CWR bouts ≥30 min provide no evidence of greater lipid use for overweight/obese individuals [23,26,41,43,45,46,48,50,53,57]. Nevertheless, it seems reasonable to suggest that studies designed to investigate the influence of overweight/obesity on exercise lipid use should involve exercise that is at least 30 min long to allow any duration-dependent increase in lipid mobilization and subsequent oxidation to occur [113]. This is also important in light of the practice of using incremental exercise with relatively short stages to assess substrate use for the work rate maintained during the stage; for example, as was done in eight included studies [42,43,51,52,54,55,58,59]. Indeed, Steffan et al. (1999) confirmed that RER changes that occur after minute three of CWR exercise at both 50% and 75% VO_2max_ resulted in significant differences at both 8 and 15 min compared to the value measured at three min and that which was measured during a 3-min stage of an incremental bout [58]. They rightly conclude that ‘steady-state’ substrate use cannot be predicted from graded-exercise tests [58].

#### 3.4.3. Intensity

Intensity profoundly influences the proportional use of substrate during physical activity. For example, in support of the ‘crossover concept’ [114], Venables et al. (2005) found that carbohydrate oxidation continued to increase from low to high work rate during incremental treadmill exercise while lipid use only did so until an intermediate intensity was encountered. Indeed, once the peak rate of lipid use was reached, lipid reliance decreased progressively until its contribution became negligible at the highest work rates (e.g., ≥~84% VO_2max_) [115]. The observation that the highest lipid-oxidation rate occurs at an intermediate intensity (e.g., ~48% VO_2max_) forms the basis for using moderate-intensity exercise to asses exercise lipid oxidation. However, the authors also observed considerable variability across the 300 participants they tested with the maximal rate occurring from 25–77% VO_2max_ [115]. Consequently, it stands to reason that exercise intensity should be assigned for such testing in a participant-specific manner. The traditional way to normalize endurance-exercise intensity is by quantifying work rate relative to VO_2max_ based on the assumption that different people exercising at the same percentage of VO_2max_ will experience similar physiological challenge. However, it is now well established that the metabolic response to a linear increase in work rate exhibits nonlinear behaviour that is unique for different individuals [116,117]. Consequently, exercise performed at the same percentage of VO_2max_ can represent markedly different physiological challenges (including the ability to satisfy the required energy turnover via lipid oxidation) for two VO_2max_-matched individuals. Indeed, assigning exercise intensity relative to VO_2max_ fails to account for other important parameters of aerobic fitness; for example, lactate threshold (LT) and the asymptote of the hyperbolic relationship between power production and time to exhaustion (i.e., the ‘critical power;’ CP), which serve as upper boundaries of the moderate- and heavy-intensity domains, respectively [116]. Importantly, Venables et al. (2005) showed that LT, which occurred at ~65% VO_2max_ (range, 45–89%) in the 300 individuals that they tested, was aligned with a higher work rate compared to the maximal lipid-oxidation rate [115]. Consequently, in lieu of a direct measurement of the maximal lipid-oxidation rate, it seems reasonable to conclude that tests designed to assess exercise lipid use should be performed at intensities below LT with work rate assigned relative to it.

Fourteen of 17 included studies that involved CWR exercise and three of eight that involved incremental exercise were designed with exercise intensity assigned relative to VO_2max_ (Table 1). Moreover, in the five other studies using incremental testing, stages were performed at the same absolute work rates for all participants. Conversely, only two studies involving CWR exercise were designed with intensity assigned relative to ‘VT’ (i.e., ‘ventilatory threshold,’ one of many terms used to describe LT [118]) and in both cases, exercise was performed ‘at VT,’ which implies that most participants were operating at an intensity that exceeded that which allowed for their maximal capacity for lipid use [23,56]. Furthermore, in one of those studies [23], the parameter was identified according to the ventilatory equivalent for CO_2_, which is the point at which respiratory compensation begins (i.e., the ‘second ventilatory threshold’ or VT_2_). Importantly, VT_2_ emerges subsequent to the initial VT (i.e., VT_1_) during incremental tests with sufficient sensitivity to reveal the ‘isocapnic region’ within which arterial partial pressure of CO_2_ is maintained despite lactic acidosis [119]. Conversely, Santiworakul et al. used the V-slope method for identification, which reveals VT_1_ (and, therefore, LT) regardless of incremental protocol [56]. These researchers found no difference in lipid oxidation at LT for obese compared to lean participants.

## 4. Materials and Methods

This systematic review was performed according to the Preferred Reporting Items for Systematic Reviews and Meta-Analysis (PRISMA) guidelines and is registered in the PROSPERO database (PROSPERO 2015:CRD42015027519).

### 4.1. Search Strategy

Two researchers independently conducted a search and selected the articles included in this review. The search, which was updated through 31 October, 2019, was performed using the databases PubMed, ProQuest, ISI Web of Science, and the Cochrane Library. Search terms were: (1.) ‘obes’ or ‘overweight:’ (2.) ‘substrate utilization’ or ‘lipid oxidation’ or ‘fat oxidation’; and (3.) ‘exercise’ or ‘fatmax’ or ‘fatomax’ or ‘lipomax.’ Limits included articles that: (1.) Appeared in a peer-reviewed journal published in English; and (2.) reported findings from an IRB-approved clinical trial on humans. Following extraction, reference lists of remaining studies were examined so that relevant articles not flagged by the search were considered.

### 4.2. Inclusion and Exclusion Criteria

Articles were included if they presented original research regarding the influence of overweight/obesity on fuel use during exercise in generally-healthy (i.e., without metabolic, cardiopulmonary, neurological or musculoskeletal disease, and/or not taking medication that might affect fuel metabolism), sedentary adults (>18 years). Accordingly, for a study to be included, participants had to be stratified into one of at least two distinct groups defined according to either Body Mass Index (BMI) or body-fat percentage. Articles were excluded if they assessed: (1.) Older adults (>65 years); (2.) individuals with chronic disease and/or exercise limitations; and (3.) physically-active individuals (e.g., recreational or competitive athletes).

### 4.3. Data Extraction and Synthesis

After selection, one author extracted the following information from included articles: Author(s), journal, publication date, sample size, participant characteristics (e.g., age, sex, BMI, body-fat percentage, maximal/peak rate of oxygen consumption; VO_2max_/VO_2peak_), criterion-exercise characteristics (e.g., mode, intensity, duration), aim(s), hypothesis(es), methodological design, primary outcomes, and additional relevant findings.

### 4.4. Quality Assessment

We combined relevant questions from three widely-accepted scales (PEDro, MINORS, NIH) to assess methodological quality of the included studies. Two researchers provided independent assessments by judging control as either absent/insufficient, present/insufficient, or present/sufficient (score = 0, 1, or 2, respectively). Scores were averaged to provide a single rating per study. Regardless of assessed quality, all studies satisfying the inclusion criteria were included in this review with scores revealed to provide perspective.

## 5. Conclusions

The contention that there is a lipid-oxidation decrement associated with overweight/obesity comes predominantly from research indicating that obese individuals demonstrate lower rates of lipid oxidation during fasting when lipid use should predominate. With respect to moderate-intensity exercise where lipid should still contribute significantly, it is difficult to reconcile how a ‘normal’ capacity could be maintained in the face of overweight/obesity if such a deficiency is present. Nevertheless, we have found that the majority of evidence indicates overweight/obese and normal-weight individuals rely on lipid to a similar extent during exercise. Moreover, there is some evidence which indicates that exercise lipid use might be increased for overweight/obese individuals possibly due to the confounding influence of alterations in source-specific lipids secondary to IR. Regardless of this distinction, the fact that overweight/obese individuals demonstrate at least normal lipid turnover during exercise contrasts the contention that the pathological accumulation of lipid in muscle and consequent presence of lipotoxic spingolipid metabolites (e.g., ceramide) that adversely affects the insulin signal transduction pathway occurs because of a lipid-oxidizing defect associated with overweight/obesity. Instead, our findings provide support for the contention that regular exercise is an important adjunct to diet for these individuals to lose body fat and maintain/restore metabolic health by using their normal capacity for lipid oxidation during exercise to ‘turn over’ potentially deleterious lipid stores on a regular basis.

## Figures and Tables

**Table 1:** Characteristics of the participants assessed, criterion exercise bout(s) used to determine fuel use, conclusions, and outcome measure(s) cited for the 24 included studies.

Study	Participants		Exercise for Assessment	Relevant Outcome Measure(s)	Lipid Use for Overweight/Obese
Ardévol et al. (1998) [42]	Female		Leg cycling		↔
	Obese: *n* = 8, age 30.0 years, BMI 32.3 ± 0.8 kg/m^2^, body fat 35.7% ± 1.1%	Control: *n* = 8, age 25.4 years, BMI 20.9 ± 0.5 kg/m^2^, body fat 23.4% ± 1.3%	Incremental O W + 30, W↑/3-min stage → T_lim_	RQ	
Balci (2012) [43]	Male		Treadmill		↔
	Overweight/Obese: *n* = 9, age 21.4 ± 0.6 years, BMI 31.6 ± 1.1 kg/m^2^, body fat 24.2% ± 1.3%	Normal Weight: *n* = 10, age 21.9 ± 0.7 years, BMI 22.6 ± 0.4 kg/m^2^, body fat 13.8% ± 0.5%	CWR (1/session) 45 min at 1 km below PTS, 45 min at 1 km above PTS, Incremental Modified Bruce Protocol	Lipid oxidation rate (g∙min^−1^), Maximal lipid oxidation rate (g∙min^−1^, mg∙kgFFM^−1^∙min^−1^, RER, VO_2_∙kg^−1^BM, %VO_2max_, HR)	
Chatzinikolaou et al. (2008) [44]	Male		Resistance		↓
	Obese: *n* = 8, age 23.4 ± 0.8 years, BMI 31.2 ± 1.0 kg/m^2^, body fat 27.7% ± 1.2%	Lean: *n* = 9, age 23.8 ± 1.2 years, BMI 23.7 ± 0.5 kg/m^2^, body fat 11.1% ± 1.4%	Circuit 3 cycles × 10 exercises w/10–12 reps/set separated by 30-s rest	RER	
Colberg et al. (1996) [45]	Male/Female		Leg cycling		↔
	Obese: *n* = 4/3, age 50 ± 3 years, BMI 35.0 ± 1.4 kg/m^2^	Lean: *n* = 3/4, age 48 ± 3 years, BMI 23.4 ± 1.0 kg/m^2^	CWR 40 min at 40% VO_2peak_	RER Lipid oxidation rate (mg∙BM^−1^∙min^−1^). Lipid energy expenditure (% total)	
Devries et al. (2013) [41]	Female		Leg cycling		↔
	Obese: *n* = 11, age 40 ± 3 years, BMI pre 34 ± 2 kg/m^2^, BMI post 34 ± 2 kg/m^2^, body fat pre 49% ± 1%, body fat post 50% ± 2%	Lean: *n* = 12, age 41 ± 2 years, BMI pre 23 ± 1 kg/m^2^, BMI post 23 ± 1 kg/m^2^, body fat pre 32% ± 2%, body fat post 32% ± 2%	CWR 60 min at 50% VO_2peak_	RER Lipid oxidation rate (mg∙BM^−1^∙min^−1^, mg∙FFM^−1^∙min^−1^)	
Ezell et al. (1999) [46]	Female		Leg cycling		↔
	Obese: *n* = 5, age 26.2 ± 2.8 years, BMI 30.0 ± 2.7 kg/m^2^, body fat 44.4% ± 1.8%	Never obese: *n* = 5, age 25.6 ± 3.5 years, BMI 20.6 ± 0.9 kg/m^2^, body fat 25.0% ± 2.8%	CWR 60 min at 60–65% VO_2peak_	RER Total lipid oxidation (g, g∙BM^−1^, g∙FFM^−1^)	
Goodpaster et al. (2002) [27]	Male		Leg Cycling		↑ ↔
	Obese: *n* = 7, age 39.3 ± 3.2 years, BMI 33.7 ± 1.1 kg/m^2^, body fat 30.3% ± 1.3%	Lean: *n* = 7, age 34.3 ± 3.3 years, BMI 23.7 ± 0.7 kg/m^2^, body fat 20.4% ± 2.3%	CWR 60 min at 50% VO_2max_	RER Lipid oxidation rate (µmol∙FFM^−1^∙min^−1^), Lipid energy expenditure (MJ, % total)	
Grams et al. (2017) [47]	Male/Female		Vacuuming/Floor Walking/Platform Stepping/Leg Cycling		↓ ↔
Hickner et al. (2001) [34]	Female		Leg cycling		↓ ↔
	Obese African American: *n* = 11, age 30.9 ± 2.2 years, BMI 38.0 ± 1.8 kg/m^2^, body fat 41.2% ± 1.3%, Obese Caucasian: *n* = 9, age 34.1 ± 2.5 years, BMI 34.8 ± 0.9 kg/m^2^, body fat 39.3% ± 2.7%	Lean African American: *n* = 7; age 28.4 ± 2.8 years; BMI 23.1 ± 1.2 kg/m^2^; body fat 25.8% ± 2.8%. Lean Caucasian: *n* = 9; age 24.7 ± 1.8 years; BMI 23.5 ± 1.0 kg/m^2^; body fat 26.4% ± 2.0%	CWR (2 in succession). 10 min at 15 W;10 min at 65% VO_2peak_	RER Lipid oxidation rate (g∙min^−1^, g∙FFM^−1^∙h^−1^)	
Horowitz et al. (2000) [28]	Female		Leg cycling (recumbent)		↑
	Obese: *n* = 5; age premenopausal. BMI 37.7 ± 0.8 kg/m^2^, body fat 48.6% ± 1.9%	Lean: *n* = 5; age premenopausal. BMI 20.9 ± 0.4 kg/m^2^, body fat 25.4% ± 1.5%	CWR 90 min at 50% VO_2peak_	Lipid oxidation rate (µmol∙FFM^−1^∙min^−1^)	
Kanaley et al. (1993) [48]	Female		Leg Cycling		↔
	Obese lower body: *n* = 11; age 36 ± 2 years; BMI 31.5 ± 0.4 kg/m^2^; body fat 50% ± 3%; Obese upper body: *n* = 13; age 36 ± 2 years. BMI 33.4 ± 0.5 kg/m^2^; body fat 48% ± 2%	Non-obese: *n* = 8; age 36 ± 1 years; BMI 22.1 ± 0.6 kg/m^2^; body fat 30% ± 1%	CWR 150 min at 45% VO_2peak_	Lipid oxidation rate (µmol∙min^−1^)Total lipid oxidation (mmol)	
Kanaley et al. (2001) [49]	Female		Treadmill		↑ ↔
	Obese lower body: *n* = 11; age 32.0 ± 1.7 years; BMI 32.5 ± 0.5 kg/m^2^; body fat 38.2% ± 0.6%; Obese upper body: *n* = 12; age 32.5 ± 1.7 years; BMI 33.5 ± 1.0 kg/m^2^; body fat 38.9% ± 0.5%	Non-obese: *n* = 8; age 35.7 ± 1.4 years; BMI 21.7 ± 1.7 kg/m^2^; body fat 20.8% ± 1.4%	CWR 30 min at 70% VO_2peak_	RER Lipid oxidation rate (µmol∙FFM^−1^∙min^−1^)	
Keim et al. (1996) [50]	Male/Female		Leg cycling		↓ ↔
	Fatter: *n* = 8/8; age 34 ± 1/29 ± 2 years; body fat 22.1% ± 0.6/36.2% ± 1.8%	Leaner: *n* = 8/8; age 29 ± 1 years/32 ± 1 years; body fat 12.4% ± 0.8/20.6% ± 0.9%	Intermittent Incremental; 30, 60, 90, 120 W/5-min stage (female); 30, 60, 90, 120, 150 W/5-min stage (male)	RER Lipid oxidation rate (mg∙FFM^−1^∙min^−1^)	
Lanzi et al. (2014) [51]	Male		Leg cycling		↑ ↓ ↔
	Obese: *n* = 16; age 34.5 ± 2.1 years; BMI 39.0 ± 1.4 kg/m^2^; body fat 42.4% ± 1.4%	Lean: *n* = 16; age 33.1 ± 1.6 years; BMI 22.9 ± 0.3 kg/m^2^; body fat 19.8% ± 1.3%	Incremental 20% PPO + 7.5%↑/6-min stage → 65% or RER = 1.0	RER Lipid oxidation rate (g∙min^−1^, mg∙FFM^−1^∙min^−1^); Maximal lipid oxidation rate (mg∙FFM^−1^∙min^−1^, %VO_2peak_, %HR_max_, RER); Maximal lipid oxidation rate zone (%VO_2peak_)	
Larsen et al. (2009) [52]	Male		Leg cycling/Arm cranking		↑ ↔
	Obese: *n* = 8; age 37 ± 2 years; BMI 32 ± 1 kg/m^2^; body fat 32% ± 1%	Lean: *n* = 7; age 43 ± 3 years; BMI 25 ± 1 kg/m^2^; body fat 23% ± 1%	Incremental (leg); 95 W + 35 W↑/5-min stage → RER = 1.0 + 35 W↑/2-min stage → T_lim_; Incremental (arm); 20 W + 15 W↑/6-min stage → 65 W + 5-min rest + 15 W↑/1-min stage → T_lim_	Lipid oxidation rate (g∙min^−1^); Maximal lipid oxidation rate (g∙min^−1^, g∙BM^−1^∙min^−1^, g∙FFM^−1^∙min^−1^, %VO_2max_)	
Melanson et al. (2009) [50]	Male/Female		Leg cycling		↔
	Obese: *n* = 4/3; age 34 ± 5/44 ± 1 years; BMI 37.2 ± 3.3/31.7 ± 2.6 kg/m^2^; body fat 38.7% ± 3.1/40.8% ± 4.4%	Lean: *n* = 4/6; age 32 ± 12/30 ± 5 years; BMI 22.7 ± 2.9/22.4 ± 1.8 kg/m^2^; body fat 21.6% ± 6.6/29.9% ± 4.2%	CWR 60 min at 55% VO_2peak_	RER	
Mittendorfer et al. (2003) [53]	Male		Leg cycling (recumbent)		↔
	Overweight: *n* = 5; age 37 ± 4 years; BMI 27 ± 1 kg/m^2^; body fat 26% ± 1%; Obese: *n* = 5; age 38 ± 2 years; BMI 34 ± 1 kg/m^2^; body fat 30% ± 1%	Lean: *n* = 5; age 31 ± 3 years; BMI 21 ± 1 kg/m^2^; body fat 16% ± 2%	CWR 90 min at 50% VO_2peak_	Lipid oxidation rate (mg∙FFM^−1^∙min^−1^)	
Mohebbi and Azizi (2011) [54]	Male		Treadmill		↓ ↔
	Obese: *n* = 10; age 22.7 ± 2.0 years; BMI 32.5 ± 2.2 kg/m^2^; body fat 29.9% ± 5.3%	Normal Weight: *n* = 12; age 22.1 ± 1.5 years; BMI 22.3 ± 1.1 kg/m^2^; body fat 14.8% ± 3.9%	Incremental 3.5 km∙h^−1^ @ 1% + 1.0 km∙h^−1^↑/3-min stage × 4 stages + 2%↑/3-min stage → RER = 1.0 + speed ↑ → T_lim_	Lipid oxidation rate (mg∙FFM^−1^∙min^−1^); Maximal lipid oxidation rate (mg∙FFM^−1^∙min^−1^, %VO_2max_); Minimal lipid oxidation rate (%VO_2max_)	
Pérez-Martin et al. (2001) [55]	Male/Female		Leg Cycling		↓
	Overweight: *n* = 15/17; age 44.0 ± 2.6/43.2 ± 2.6 years; BMI 32.1 ± 1.4/29.6 ± 0.9 kg/m^2^; body fat 32.7% ± 1.4/41.6% ± 0.9%	Control: *n* = 11/15; age 36.2 ± 3.7/41.1 ± 3.3 years; BMI 23.0 ± 0.6/23.0 ± 0.4 kg/m^2^; body fat 18.3% ± 1.5/25.9% ± 0.8%	Incremental 20% WR_max(est)_ + 10% WR_max(est)_ ↑/6-min stage ×4 stages	RER Lipid oxidation rate (mg∙min^−1^, mg∙FFM^−1^∙min^−1^); Power at maximal lipid oxidation rate (%WR_max(est)_, W); Crossover point (%WR_max(est)_, W, HR)	
Santiworakul et al. (2014) [56]	Male		Mode not stated		↔
	Obese: *n* = 10; age 25.6 ± 3.9 years; BMI 31.9 ± 2.5 kg/m^2^; body fat 35.9% ± 5.1%	Lean: *n* = 10; age 25.7 ± 4.0 years; BMI 21.6 ± 1.2 kg/m^2^; body fat 19.9% ± 8.1%	CWR *x* min at VT* (*x* = time to 300 kcal expenditure)	Lipid energy expenditure (% total, kcals)	
Slusher et al. (2015) [57]	Male/Female		Treadmill		↔
	Obese: *n* = 11; age 22.9 ± 1.6 years; BMI 35.7 ± 4.2 kg/m^2^	Normal weight: *n* = 11; age 23.3 ± 2.2 years; BMI 22.0 ± 1.6 kg/m^2^	CWR 30 min at 75% VO_2max_	Lipid oxidation rate (g∙min^−1^)	
Steffan et al. (1999) [58]	Female		Treadmill		↔
	Obese: *n* = 20; age 29.8 ± 1.3 years; BMI 31.0 ± 1.7 kg/m^2^; body fat 41.0% ± 1.5%	Normal weight: *n* = 15; age 25.1 ± 1.1 years; BMI 22.1 ± 0.7 kg/m^2^; body fat 26.1% ± 0.9%	Incremental Modified Bruce Protocol; CWR (1/session) 15 min at 50% VO_2max_15 min at 75% VO_2max_	RER	
Thyfault et al. (2004) [26]	Female		Leg cycling		↔
	Obese: *n* = 10; age 38.9 ± 1.9 years; BMI 40.8 ± 1.7 kg/m^2^	Lean: *n* = 7; age 38.6 ± 2.3 years; BMI 22.6 ± 0.8 kg/m^2^	CWR 60 min at 50% VO_2max_	RER Lipid oxidation rate (µmol∙BM^−1^∙min^−1^)	
Wong et al. (2006) [23]	Male		Leg cycling		↔
	Obese: *n* = 7; age 36.1 ± 3.4 years; BMI 31.9 ± 3.8 kg/m^2^; body fat 32.2% ± 5.7%	Lean: *n* = 6; age 34.5 ± 2.6 years; BMI 21.7 ± 1.7 kg/m^2^; body fat 15.4% ± 1.0%	CWR 30 min at VT**	RER	

BM: Body mass; BMI: Body mass index; CWR: Constant-work-rate exercise; FFM: Fat-free mass (pre, prior to 12-week training intervention; post following 12-week training intervention); PPO: Peak power output; PTS: Preferred walk-run transition speed; RER: Respiratory exchange ratio; RQ: Respiratory quotient; T_lim_: Limit of tolerance; VO_2max_: Maximal rate of oxygen consumption; VO_2peak_: Peak rate of oxygen consumption; VT: Ventilatory threshold (*, identified by V-slope method; ** identified by ventilatory-equivalent-for-CO_2_ method); WR_max(est)_: Maximal work rate estimated according to prediction equations.

**Table 2:** Conclusion drawn from eight of 24 included studies that returned equivocal findings based on multiple outcome measures, populations, and/or exercise characteristics that were assessed.

Determining Factor	Study	O < NW	O = NW	O > NW	Qualifications
Exercise duration (min)	Kanaley et al. 2001 [49]		15	30	For lipid oxidation rate (LBO and UBO) or RER (UBO)
Exercise intensity (%VO_2peak/max_)	Hickner et al. 2001 [34]	65	~40		For lipid oxidation rate for Caucasians; O < NW (RER) for Caucasians at both intensities and O = NW (lipid oxidation rate, RER) for AA at both intensities
	Keim et al. 1996 [59]	40–60	30		For men; O = NW at all intensities for women
	Lanzi et al. 2014 [51]	-	60–85	20–55	For RER
		85	50–80	20–45	For lipid oxidation rate (g∙min^−1^)
		65–85	35–60	20–30	For lipid oxidation rate (mg∙kgFFM^−1^∙min^−1^)
	Mohebbi and Azizi 2011 [54]	60–80	20–50		For AM and PM
Exercise mode	Grams et al. 2017 [47]	Platform stepping	Vacuuming Floor Walking Leg cycling		For women; O < NW for men for stepping and cycling (lipid oxidation rate) or all four activities (% lipid energy)
	Larsen et al. 2009 [52]		Leg cycling	Arm cranking	For fatmax % VO_2max_; O = NW for Fatmax g∙min^−1^, mg∙kgBM^−1^∙min^−1^, mg∙kgFFM^−1^∙min^−1^
Exercise work rate (W)	Keim et al. 1996 [59]	120	30–90, 150		For men
	Lanzi et al. 2014 [51]	150	75–150	30–60	For RER
			90–135	30–75	For lipid oxidation rate
Outcome measure	Goodpaster et al. 2002 [27]		Lipid energy (MJ); Lipid oxidation rate (µmol∙FFM^−1^∙min^−1^)	Lipid energy (%) RER	*p* = 0.08
	Grams et al. 2017 [47]	Lipid energy (%); Lipid oxidation rate (kcal∙min^−1^); Lipid energy (%)	Lipid oxidation rate (kcal∙min^−1^); Peak lipid oxidation rate (kcal∙min^−1^, mg∙kgFFM^−1^∙min^−1^, %VO_2max_); Peak lipid oxidation rate (kcal∙min^−1^, mg∙kgFFM^−1^∙min^−1^, %VO_2max_)		For walking (men); For stepping (both sexes) and cycling (men); For stepping (both sexes) and vacuuming, walking, cycling (men)
	Hickner et al. 2001 [34]	RER	Lipid oxidation rate (g∙min^−1^, g∙FFM^−1^∙h^−1^)		For obese Caucasian and AA v. lean Caucasian
	Kanaley et al. 2001 [49]		RER	Lipid oxidation rate (µmol∙FFM^−1^∙min^−1^)	For LBO at 30 min
	Lanzi et al. 2014 [51]	Lipid oxidation rate (g∙min^−1^); Lipid oxidation rate (mg∙FFM^−1^∙min^−1^); Maximal lipid oxidation rate (%VO_2peak_, %HR_max_, RER) and zone (%VO_2peak_)	RER RER Maximal lipid oxidation rate (mg∙FFM^−1^∙min^−1^)		At 150 W and 85% VO_2peak_At 65–85% VO_2peak_
	Larsen et al. 2009 [52]		Lipid oxidation rate (g∙min^−1^); Maximal lipid oxidation rate (g∙min^−1^, g∙BM^−1^∙min^−1^, g∙FFM^−1^∙min^−1^)	Maximal lipid oxidation rate (%VO_2max_)	For arm cranking
	Mohebbi and Azizi 2011 [54]	Lipid oxidation rate (mg∙FFM^−1^∙min^−1^); Maximal lipid oxidation rate (%VO_2max_); Maximal lipid oxidation rate (%VO_2max_)	Maximal lipid oxidation rate (mg∙FFM^−1^∙min^−1^, %VO_2max_; Lipid oxidation rate (mg∙FFM^−1^∙min^−1^); Maximal lipid oxidation rate (mg∙kgFFM^−1^∙min^−1^); Minimal lipid oxidation rate (%VO_2max_)		For lipid oxidation rate at 60–80% VO_2max_ For lipid oxidation rate at 20%–50% VO_2max_
Participant fat deposition	Kanaley et al. 2001 [49]		LBO	UBO	For RER at 30 min; O < NW for LB and UB Obese for lipid oxidation rate at 30 min
Participant race	Hickner et al. 2001 [34]	Caucasian O	AA O		For cycling at 15 W (RER, lipid oxidation rate) and 65%VO_2peak_ (lipid oxidation rate)
Participant sex	Grams et al. 2017 [47]	Male O	Female O		For cycling (% lipid energy, lipid oxidation rate) and vacuuming, walking (% lipid energy)
	Keim et al. 1996 [59]	Male O	Female O		For cycling at 40–60% VO_2max_; O = NW for men and women at 30% VO_2max_

AA: African American; FFM: Fat-free mass; HR: Heart rate; LBO: Lower body; AM: Morning assessment; NW: Normal weight; O: Overweight/obese; PM: Evening assessment; RER: Respiratory exchange ratio; UBO: Upper body; VO_2max_: Maximal rate of oxygen consumption; VO_2peak_: Peak rate of oxygen consumption.

**Table 3:** Scores for general methodological quality for the 24 included studies (0 = absent/insufficient, 1 = present/insufficient, 2 = present/sufficient).

Study	Aim Clearly Stated and Defined	Eligibility and Inclusion Criteria Explained	Study Population Clearly Specified and Defined	Sample Size Justification Provided	Participants Recruited from Same or Similar Population	Independent Variable Clearly Defined, Valid and Reliable	Dependent Variable Clearly Defined, Valid, and Reliable	Avg Score
Ardévol et al. 1998 [42]	2.0	2.0	2.0	0.0	0.5	1.5	2.0	1.43
Balci 2012 [43]	2.0	2.0	2.0	0.0	0.5	2.0	2.0	1.50
Chatzinikolaou et al. 2008 [44]	2.0	1.0	2.0	0.0	0.5	1.5	2.0	1.29
Colberg et al. 1996 [45]	2.0	2.0	2.0	0.0	0.5	2.0	2.0	1.50
Devries et al. 2013 [41]	2.0	2.0	2.0	0.0	1.0	2.0	2.0	1.57
Ezell et al. 1999 [46]	2.0	2.0	2.0	0.0	0.5	1.5	2.0	1.43
Goodpaster et al. 2002 [27]	2.0	2.0	2.0	1.0	1.5	2.0	2.0	1.79
Grams et al. 2017 [47]	2.0	2.0	2.0	0.5	1.0	2.0	2.0	1.64
Hickner et al. 2001 [34]	2.0	1.5	2.0	0.0	0.5	1.5	2.0	1.36
Horowitz et al. 2000 [28]	2.0	1.5	2.0	2.0	1.0	2.0	2.0	1.79
Kanaley et al. 1993 [48]	2.0	1.5	2.0	0.0	0.0	2.0	2.0	1.36
Kanaley et al. 2001 [49]	2.0	2.0	2.0	0.0	2.0	2.0	2.0	1.71
Keim et al. 1996 [59]	2.0	2.0	2.0	0.0	0.5	2.0	2.0	1.50
Lanzi et al. 2014 [51]	2.0	2.0	2.0	0.0	1.5	2.0	2.0	1.64
Larsen et al. 2009 [52]	2.0	1.5	2.0	0.0	0.5	2.0	2.0	1.43
Melanson et al. 2009 [50]	2.0	2.0	2.0	0.0	2.0	2.0	2.0	1.71
Mittendorfer et al. 2003 [53]	2.0	1.0	2.0	0.0	0.5	2.0	2.0	1.36
Mohebbi and Azizi 2011 [54]	2.0	2.0	2.0	0.0	1.0	2.0	2.0	1.57
Pérez-Martin et al. 2001 [55]	2.0	1.0	1.5	0.0	1.0	2.0	2.0	1.36
Santiworakul et al. 2014 [56]	2.0	1.5	1.5	2.0	0.0	1.5	2.0	1.50
Slusher et al. 2015 [57]	2.0	2.0	2.0	0.0	0.0	2.0	2.0	1.43
Steffan et al. 1999 [58]	2.0	1.0	2.0	0.0	1.0	2.0	2.0	1.43
Thyfault et al. 2004 [26]	2.0	2.0	2.0	0.0	0.0	2.0	2.0	1.43
Wong et al. 2006 [23]	2.0	1.5	2.0	0.0	0.5	2.0	2.0	1.43

## Abbreviations

AA	African American
Akt	Protein kinase B
AM	Morning assessment
BM	Body mass
BMI	Body mass index
CRF	Cardiorepiratory fitness
CWR	Constant-work-rate exercise
DAG	Diacylglycerols
FATmax	Maximal rate of lipid oxidation
FFA	Free fatty acid
FFM	Fat-free mass
FQ	Food quotient
HR	Heart rate
IMTG	Intramuscular triglyceride
IR	Insulin resistance
IRS1	Insulin receptor substrate 1
LBO	Lower body obese
PKR	Protein kinase R
PPO	Peak power output
PTS	Preferred walk-run transition speed
RER	Respiratory exchange ratio
RQ	Respiratory quotient
T_lim_	Limit of tolerance
T2D	Type 2 diabetes
VO_2max_	Maximal rate of oxygen consumption
VO_2peak_	Peak rate of oxygen consumption
VT	Ventilatory threshold
WHO	World Health Organization
WR_max(est)_	Maximal work rate estimated according to prediction equations
NW	Normal weight
O	Overweight/obese
PM	Evening assessment
UBO	Upper body obese
